# Views of healthcare professionals regarding care of children with intellectual disability: A qualitative study in Vhembe district, Limpopo province

**DOI:** 10.4102/ajod.v15i0.1797

**Published:** 2026-01-21

**Authors:** Ndidzulafhi S. Raliphaswa

**Affiliations:** 1 Department of Advanced Nursing Science, Faculty of Health Sciences, University of Venda, Thohoyandou, South Africa

**Keywords:** care, support, healthcare professionals, intellectual disability, South Africa, views

## Abstract

**Background:**

Intellectual disability is a significant concern to both mothers and healthcare professionals. This is because of the support and care needed by these children. Healthcare professionals face various challenges while providing support to mothers of children with intellectual disabilities.

**Objectives:**

The study focused on exploring the views of healthcare professionals regarding the healthcare of children with intellectual disabilities in the Vhembe district, Limpopo Province.

**Method:**

A qualitative, explorative and descriptive design was used to explore the views of 15 participants who were selected purposively. In-depth individual interviews were used to collect data from the healthcare professionals who were working in paediatric wards. The data were analysed using Tesch’s eight steps. Measures to ensure trustworthiness were adhered to throughout the study.

**Results:**

This study revealed that a shortage of healthcare professionals, especially nurses, doctors, physiotherapists and occupational therapists, had a negative impact when providing healthcare for a child with intellectual disability. Moreover, a lack of experienced personnel to care for these children and a lack of training were found to be challenges faced by healthcare professionals.

**Conclusion:**

Increasing the number of healthcare staff and providing training to them in intellectual disability care to enhance knowledge, competency, and skills were found to be effective strategies that help provide the needed emotional, psychological, and social support for children with intellectual disabilities and their mothers.

**Contribution:**

This study adds to the literature by highlighting the vital role played by nurses, doctors, physiotherapists and occupational therapists in supporting children with intellectual disabilities and their mothers.

## Introduction

When children with intellectual disabilities are sick, they rely on healthcare professionals for care, just like any other child. Healthcare professionals must ensure that children with intellectual disabilities receive effective care and empirically supported treatments. (Raliphaswa 2018). It is important for healthcare professionals to acknowledge and respect the needs and desires of parents regarding their children, as these matters are often sensitive and deeply personal (Oulton, Sell & Gibson 2020). Despite the extra healthcare needed by children with intellectual disability, they are usually accommodated in hospital settings among children without intellectual disability. The current shortage of staff presents challenges in ensuring adequate support for children with intellectual disabilities. To enhance their care, it is essential that we prioritize increasing staffing levels, as all children benefit from attentive monitoring and support. Implementing strategies to attract and retain qualified professionals will help us provide the close care that every child deserves (Ong et al. 2022). Furthermore, healthcare professionals play a crucial role in delivering safe and high-quality care to children, which is essential for fostering the overall health and well-being of both mothers and their children. This involves not only addressing immediate medical needs but also providing comprehensive support that includes preventive measures, education on healthy practices, and guidance on nutritional and developmental needs. By ensuring a supportive and nurturing environment, healthcare providers can significantly contribute to positive health outcomes for families during these critical early years (Hemsley et al. 2016).

Parents, especially the mothers, of children with intellectual disabilities have an increased risk for parenting stress and psychological distress resulting from multiple roles they play in caring for their children (Feizi et al. 2014). These roles include, among other things, medication and treatment management, monitoring the child’s health and facilitating access to healthcare services. These responsibilities are time-consuming for mothers and might detract from proper care to other children as she might not have enough free time (Geuze, Goossensen & Schrevel 2022). Healthcare professionals have observed a growing complexity in caregiving situations, particularly when extended family members, such as grandparents and spouses, become involved. This trend is especially pronounced when these relatives rely on the mother as their primary caregiver. The increasing demands on her time and energy can lead to heightened stress and emotional strain, as she navigates the challenges of coordinating care for multiple family members who depend on her support and nurturing. This intricate web of responsibilities can hinder her ability to provide adequate attention to each person’s needs, further complicating the family dynamics and potentially impacting the overall well-being of all involved. A study conducted by Warmingham, Rogosch and Cicchetti (2020) found that children are often mistreated when their mother shows signs of mental distress. Addressing neglect in physical and medical care is crucial, as it enables individuals to effectively utilise available medical and welfare support services. By improving these areas, we can enhance access to essential resources and promote overall well-being. The demand for more specialists, especially psychologists, was seen to be a priority to support the mental health needs of both the children and their parents (Kobayashi, Inagaki & Kaga 2012). It is essential for healthcare professionals to empower mothers by providing them with comprehensive and accurate information about the healthcare needs of their children with intellectual disability. By ensuring access to this essential knowledge, they can support families in making informed decisions for their child’s well-being. To alleviate anxiety and psychological stress, it is important to involve their siblings as they are part of the family who interact with their sibling with intellectual disability. This might assist in enhancing trust in healthcare professionals as they might feel supported and cared for (Jansen, Van der Putten & Vlaskamp 2017).

Moreover, healthcare professionals sometimes experience difficulties in maintaining good relations or communicating with parents because of a number of factors, including work overload (Fearnley & Boland 2017). Unfortunately, failure to adequately interact with parents results in information being missed, as caregivers or parents have useful and important information to share with the healthcare professionals (Charles 2020). Therefore, it was against this background that this study sought to explore and describe the views of healthcare professionals regarding the healthcare of children with intellectual disability in the Vhembe district, Limpopo Province.

## Research methods and design

### Study design

A qualitative, contextual, explorative and descriptive design was chosen in this study in order to explore the views of healthcare professionals regarding the healthcare of children with intellectual disabilities. Data were collected from the healthcare professionals about their views when caring for a child with intellectual disabilities in selected healthcare institutions of Vhembe district, Limpopo Province.

### Study setting

The study was conducted in seven selected healthcare institutions of Vhembe district in Limpopo province, South Africa. Only those institutions with paediatric wards were selected. Limpopo Province is situated in the North-Eastern corner of South Africa and shares borders with Botswana, Zimbabwe, and Mozambique.

### Population

The target population was healthcare professionals caring for children with intellectual disability who were admitted to the paediatric wards of the selected institutions. Non-probability purposive sampling was used to select the participants. Only participants 21 years and above were selected as long as they fell within the specified categories of healthcare professionals, including those who provide special health needs like physiotherapists, occupational therapists, as they had enough information to share regarding care needed for children with intellectual disability. Before being selected, the participants were given a thorough explanation of the details of the study, and informed consent was obtained.

### Sample size

The study sample comprised 15 healthcare professionals. Of the 15 healthcare professionals, two were physiotherapists, three were occupational therapists, two were doctors, one psychologist and seven were nurses. These participants were included in this study because they play a major role in addressing emotional, psychological, and other health problems related to mothers and their children with intellectual disability.

### Instrument and data collection method

Unstructured, in-depth individual interviews were used in person to obtain full and rich information about the views of healthcare professionals when providing healthcare for children with intellectual disabilities. One central question was posed: ‘May you kindly share your views regarding care of children with intellectual disability’, and follow-up questions were asked based on the participant’s responses. Furthermore, a pre-test was conducted on two participants before the actual study was conducted to assist in identifying any challenges or difficulties that could occur during data collection. For example, checking the researcher’s ability to ask questions and probe in such a way that the participants will be able to understand the question and answer relevantly. This is important because the researcher is an instrument for data collection. However, there were no questions that required alterations after the pre-test. Participants who were part of the pre-test were not included in the main study.

Interviews were conducted in English, audio-recorded, transcribed verbatim, and stored electronically with password protection during analysis. English was chosen because participants could express themselves effectively as professionals. The interviews lasted for 30 to 45 min. Field notes, including non-verbal cues, were also taken during interviews. Data were saturated at the 10^th^ participant. However, to ensure that no new information was coming in, the researcher went on to add another 5 participants. Transcripts were returned to the participants for their comments and to verify that the information captured was the true reflection of what they said.

### Data analysis

The collected data were analysed using Tesch’s eight steps as it is useful for unstructured data, as described by Creswell (2017). The eight steps involved are: getting a sense of the whole, picking one document at a time, making a list of all topics, taking the list of topics and going back to the data, turning topics into categories, making a final decision, assembling the data, and recording the data. This involved transcribing information verbatim from the audio recorder. After transcribing, the researcher repeatedly read each transcript to familiarise herself with the data, simultaneously sorting the similar and different ideas. The analysis was done by the researcher with the assistance of supervisors. An independent co-coder cross-checked the data for categories, sub-themes, and themes to determine if they were linked to what the participants said during the interviews. An agreement was reached after discussion to confirm the themes and sub-themes. Then the qualitative data were classified by looking for categories. Finally, themes and sub-themes were developed and refined. The interpretation of findings was based on the integration of various literature regarding the topic of study.

### Ethical considerations

Ethical clearance to conduct this study was obtained from the University of Venda research ethics committee (No. SHS/16/PDC/07/2804). All participants provided written informed consent to participate. Ethical principles were adhered to; for example, confidentiality and anonymity were maintained by substituting participant names with codes. Permission was also sought from the provincial Department of Health and obtained. However, the departmental permission required that Health care professionals (HCP) routines not be disrupted by participation in the research study. The researcher had to make appointments with the healthcare professionals on duty and agree on a specific time that would be more suitable for the interviews. Participants were informed about the purpose of the study and its significance. They were also informed that the interviews would be recorded as well as the rationale for this was indicated before they gave consent. All ethical principles like anonymity, confidentiality and voluntary participation were adhered to.

## Results

The participants comprised 15 healthcare professionals: 2 doctors, 7 nurses, 2 physiotherapists, 1 psychologist and 3 occupational therapists. The age distribution of the respondents is encouragingly varied, reflecting a broad range of perspectives. Specifically, there were two respondents aged 21 years to 25 years, four aged 26 years to 30 years, four aged 31 years to 35 years, and five aged over 35 years. Table 1 shows the demographic details of the participants.

**TABLE 1:** Participants’ demographic data.

Participant number	Age (years)	Category	Working experience (years)	Speciality
1	22	Enrolled nurse	1	None
2	27	Enrolled nurse	3	None
3	32	Occupational therapist	2	None
4	25	Physiotherapist	3	None
5	26	Occupational therapist	6	None
6	33	Doctor	4	None
7	53	Professional nurse	10	None
8	35	Auxiliary nurse	8	None
9	28	Occupational therapist	3	None
10	31	Psychologist	4	None
11	36	Doctor	5	Paediatrician
12	40	Enrolled nurse	2	None
13	30	Physiotherapist	2	None
14	56	Professional nurse	2	None
15	45	Professional nurse	10	Paediatric nurse trained

The study findings revealed four themes. Theme one was ‘shortage of staff’, theme two was ‘lack of knowledge impacting proper communication’, theme three was ‘need for supportive care’ and theme four was ‘increased risk for parental stress and psychological distress’. These themes are outlined in Table 2.

**TABLE 2:** Themes.

Theme	Topic
1	Shortage of staff
2	Lack of knowledge impacting proper communication
3	Need for supportive care
4	Increased risk for parental stress and psychological distress

### Theme 1: Shortage of staff

Shortage of staff was a challenge, especially nurses in paediatric units, as children, whether they have a disability or not, require more attention than adults who are able to take care of themselves.

One participant said:

‘We are short-staffed in this unit, and it is difficult to give full care to all children. Children need constant observation and monitoring because those who can run around can cause medicolegal hazards to themselves, to those who are critically ill and to those with intellectual disabilities.’ (P1)

Follow-up questions were done with Participants 2 and 14.

One participant added by saying:

‘Shortage of staff has been a daily song. People are resigning, retiring, and even dying, but they are not being replaced. You know, I am on night shift, we are only three, and we are not coping because sometimes the ward becomes full of children who cannot feed or even bathe themselves. Despite this, children are not allowed to do any task on their own without supervision.’ (P12)

Another participant said:

‘We have been complaining about a shortage of staff as it compromises quality patient care. Mothers of children with intellectual disability are to be supported at all costs but one cannot really do the best with this shortage …’ (P7)

From the quotation above, it is clear that the shortage of staff is indeed a concern to nurses. Nurses working in the paediatric ward need to be well-staffed as compared to other wards. This is because the physical safety of children, feeding and treatment management are of vital importance; if close monitoring is not done, children are at risk of injuries caused by electric shock, burns, drinking illegal fluids, and hurting others, especially the bedridden ones.

### Theme 2: Lack of knowledge impacting proper communication

Healthcare professionals felt that they had inadequate knowledge, while also finding that mothers also lacked knowledge on intellectual disability. This impeded communication, with some healthcare professionals sharing that they felt ashamed about being ill-equipped to provide the information needed by mothers. One participant shared:

‘You know, knowledge is power. We are trained to a certain limit, and some diseases these children are admitted with are completely new to me. Sometimes I feel ashamed when a poor mother asks me a question, as I won’t be able to give a satisfactory answer.’ (P1)

Healthcare professionals noted the need for training, especially for nurse participants:

‘It is very important to be trained to gain more knowledge on childhood illnesses and how to take care of them.’ (P6)

While they often regarded more qualified and trained colleagues as possessing superior knowledge, there were instances where these colleagues did not share the same level of experience. This highlights an opportunity for growth and knowledge sharing among all team members.

This was supported by one participant, who said:

‘Lack of knowledge results in poor communication because one cannot disseminate information to someone when you do not have that knowledge. It is very important to be trained to gain more knowledge on childhood illnesses and how to take care of them.’ (P6)

She further said:

‘All mothers depend on us for knowledge so that they get empowered to increase the little knowledge they have.’ (P6)

Some healthcare professionals blamed themselves for lacking knowledge, especially the auxiliary and enrolled nurses. They felt that professional nurses are the best people to answer some questions because they have advanced knowledge that they do not have. Professional nurses who are not trained in paediatric care also felt incompetent, especially in providing specialised care such as tube feeding a child with intellectual disability.

### Theme 3: Need for supportive care

The majority of participants in this study verbalised the need for providing supportive care to mothers of children with intellectual disability. This was in relation to what they observed when they interacted with mothers and children during follow-up visits. They shared:

‘We, as healthcare professionals need to really appreciate these mothers rather than always blaming them for missing appointments. We must listen to them and give them a chance to explain their challenges and fears. Then we will know how to assist them and provide relevant support they need. When we encourage the family members to give full support, let them see and copy from us. Let’s show them “Ubuntu” and do as we are expected to do.’ (P11)

This was supported by two participants who said:

‘Ok … it is better to review these children the same day all, of us if it is necessary. Giving them different review dates is expensive because some of our patients are from very far away and they do not have reliable transport or own cars. Besides, those mothers who are working must not always be absent from work, if there is a way to reduce this, unless there is something urgent or special that needs to be done. Let’s support and think for them that they are also human beings with feelings like any other mother. I think we will be doing justice to them.’ (P4)

‘If we give them the necessary support, they will see a reason to come for follow-up visits. Some mothers discontinue the therapy needed by a child because their concerns are not entertained, or no one is willing to assist.’ (P9)

Supportive care includes counselling and scheduling follow-up visits on the same day, to reduce the financial burden of coming to the hospital on different occasions. Mothers are the ones to be given support because they play a major role in providing care to their children with intellectual disabilities.

### Theme 4: Increased risk of parental stress and psychological distress

Participants in this study revealed that caring for children with intellectual disability causes stress and frustration in mothers if they are not fully supported. This was supported by a participant who said:

‘These mothers sometimes just burst out even if there is nothing wrong you did to them. I think there are not coping with the child’s condition. They really need support from home and from us as healthcare professionals … *eish*.’ (P15)

Two other participants had this to say:

‘It is not easy really. When you instruct the mother to assist the child with certain simple activities, most of them don’t do as you say. It is like they do not understand and looks like you are giving them extra job. I am trying my best, but at times, I am not winning. *Yooh* … you can feel for this mothers … shame.’ (P5)

‘… these mothers look frustrated and stressed because they seem not to be getting enough support from the healthcare professionals.’ (P2)

Another participant added by saying:

‘If we work together and show willingness to assist these mothers, they will definitely improve their behaviour and attitudes towards us as healthcare professionals. The more we accept them and their children, the more they will see light in the dark.’ (P13)

Healthcare professionals like occupational therapists and professional nurses noted that mothers of children with intellectual disabilities are so stressed and need emotional and psychological support from healthcare professionals. Therefore, it is crucial that healthcare professionals should avail themselves to provide the needed supportive care to them.

## Discussion

The research question sought to examine healthcare professionals’ views on the care of children with intellectual disability. It was found that there was a need for more support for mothers of children with ID who experienced distress and inadequate support in the hospital settings (Douglas, Redley & Ottmann 2016). This suggests that support, especially psychological or mental health support for mothers, needs improvement because poor maternal mental health can have negative impacts on their children with intellectual disability (Rydzewska et al. 2021). Children with intellectual disabilities need a multidisciplinary team to review them. This is because they face more challenges than any other type of disability (Whitehead et al. 2021). This study revealed that the care of children with intellectual disability may be negatively impacted by staff shortage, especially professional nurses in the included facilities. Moreover, the few staff members become overwhelmed with a multitude of responsibilities, all aimed at providing the best care and support for the children. Healthcare professionals noted that there was not enough staff despite their complaints, and the existing staff are not adequately trained (Kleintjes et al. 2020).

This was confirmed by one mother who indicated that it took her a month to consult a psychologist because there were many patients waiting on the list for consultation. This study’s findings concur with the study done by Shamsi and Peyravi (2020), who indicated that a shortage of staff is related to difficulties in providing quality services. Institutions with well-staffed professionals are able to render comprehensive services, including a full examination or assessment to exclude any abnormalities, which can be done through proper history information. It is important to assess the parent’s mental health needs to see if she is coping or not. Often mothers present with symptoms of depression, anxiety and psychological distress (Feizi et al. 2014). If the mental health of a mother is disturbed, it will also adversely affect her child.

The study highlighted that most participants had relatively limited professional experience in the field. This suggests a need for training and development within the workforce. It was important to know the working experience of healthcare professionals because working with children who have intellectual disability requires someone who is knowledgeable and competent. Moreover, healthcare professionals with little or no experience pose a negative impact on mothers as they lack the continuous support they deserve from them (Shakespeare et al. 2019). Additionally, this indicates that training and developing the staff by the institution’s management is very crucial to prevent service failures (Smith, Papadakis & Munnik 2023). Therefore, the healthcare professionals should be more competent, self-assured, and committed to the institution. This will make the patients happier and more satisfied (Al Otaibi et al. 2022).

Healthcare professionals indicated that there is a need for empathetic support for mothers of children with intellectual disabilities. Healthcare professionals also indicated that there is a need for them to provide ongoing support and encouragement to mothers of children with intellectual disabilities. Furthermore, healthcare professionals shared that mothers need support and are sometimes unsupported by not being listened to or their circumstances not being taken into account.

Healthcare professionals noted that parents experienced distress due to a lack of support and knowledge. This was because of inadequate communication between mothers and healthcare professionals. Improper communication hinders the child’s progress as mothers looked anxious about the child’s condition, and they could not provide proper care to their children. Effective healthcare provision relies on the communication skills of healthcare professionals, which lead to positive outcomes such as reduced anxiety, guilt, pain, and disease symptoms (Sharkiya 2023). Occupational therapists, physiotherapists, nurses, doctors and psychologists are in demand to support the mental health needs of both parents and their children with intellectual disability (Gilson et al. 2018). Xu (2020)’s study showed results similar with the findings of this study, which revealed that engaging children and their families by the healthcare professionals is an important factor for supporting families of children with intellectual disabilities. Some nurses in this study indicated that mothers wait for too long before they can be seen by the members of the multidisciplinary team, such as psychologists, to see either the child or both of them. This was supported by a study indicating that some parents discontinue therapy due to long waiting lists, unmet expectations for therapy, and beliefs about therapeutic processes that they feel are unhelpful (Gopalan et al. 2010). Therefore, ongoing counselling and continuous support by the psychologist play a vital role in the mental health of a mother, including the family (Stancin & Perrin 2014). Furthermore, engaging families in mental health treatment remains a serious challenge in most health institutions, despite ongoing advances in evidence-based treatments (Peters-Corbett et al. 2024). Therefore, the support needs of mothers of children with intellectual disability should be taken as a priority as this affects the lives of all family members.

This study’s findings revealed that healthcare professionals observed that mothers of children with intellectual disability experienced much stress and distress. Stress can arise from a child’s behaviour and daily living skills (Peer & Hillman 2014). This was also confirmed by the study conducted by Raliphaswa, Maluleke and Netshikweta (2022) and Modula and Chipu (2024) who indicated that healthcare professionals must work with parents to reduce their stress in order to optimise outcomes for children with disabilities and their families. Research suggests that the stress and psychological distress parents experience during their early years can play a significant role in helping them develop coping strategies for the future. The study findings are supported by a study conducted by Yesilkaya and Magallón-Neri (2024), which indicated that parents of children with autism spectrum disorder and developmental disorders demonstrated increased parenting-related stress compared to parents of typically developing children. This is because of isolation, discrimination and stigma (Broady, Stoyles & Morse 2020; Mkabile & Swartz 2020) associated with the disability. Therefore, continuous support is needed in order to improve a child’s functioning and decrease parenting-related stress as they are faced with numerous challenges (Lee-Maturana, Matthewson & Dwan 2020; Raliphaswa 2018). To effectively support the healthcare needs of children with intellectual disabilities, it is essential to have a multidisciplinary team that can provide comprehensive and holistic care. This was seen to be a critical period for parents of children with intellectual disabilities (Weiss et al. 2021). Therefore, long-term strategies can assist in reducing health problems of mothers and caregivers of children with intellectual disability (Molefe 2024).

## Conclusion

To effectively support the healthcare needs of children with intellectual disabilities, it is essential to have a multidisciplinary team that can provide comprehensive and holistic care.Therefore, for the healthcare professionals to provide the needed care, they must also be empowered and given the necessary support by the management. This study recommends having enough experienced staff in paediatric units; as providing healthcare for children with intellectual disabilities can become overwhelming. This can be achieved through training more staff. Future studies are also necessary to explore the views of caregivers on the support needed for them to provide proper care to their children with intellectual disability.
